# Transgenically-expressed secretoglobin 3A2 accelerates resolution of bleomycin-induced pulmonary fibrosis in mice

**DOI:** 10.1186/s12890-015-0065-4

**Published:** 2015-07-16

**Authors:** Yan Cai, Mitsuhiro Yoneda, Takeshi Tomita, Reiko Kurotani, Minoru Okamoto, Taketomo Kido, Hiroyuki Abe, Wayne Mitzner, Arjun Guha, Shioko Kimura

**Affiliations:** Laboratory of Metabolism, National Cancer Institute, National Institutes of Health, Bethesda, MD 20892 USA; Biochemical Engineering, Graduate School of Science and Engineering, Yamagata University, Yonezawa, Yamagata 992-8510 Japan; Bloomberg School of Public Health, Johns Hopkins University, Baltimore, MD 21205 USA; Department of Medicine, Pulmonary Center, Boston University School of Medicine, Boston, MA 02118 USA; Laboratory of Liver Diseases, National Institute on Alcohol Abuse and Alcoholism, National Institutes of Health, Bethesda, MD 20892 USA; Department of Pharmacology, Tokyo Women’s Medical University, Tokyo, 162-8666 Japan; Department of Veterinary Immunopathology, School of Veterinary Medicine, Rakuno Gakuen University, Ebetsu, Hokkaido 069-8501 Japan; Laboratory of Cell Growth and Differentiation, Institute of Molecular and Cellular Biosciences, The University of Tokyo, Tokyo, 113-0032 Japan

**Keywords:** Secretoglobin, SCGB, SCGB3A2, Bleomycin-induced pulmonary fibrosis model, Spontaneous resolution of bleomycin-induced pulmonary fibrosis, Transgenic mouse

## Abstract

**Background:**

Secretoglobin (SCGB) 3A2, a cytokine-like secretory protein of small molecular weight, is predominantly expressed in airway epithelial cells. While SCGB3A2 is known to have anti-inflammatory, growth factor, and anti-fibrotic activities, whether SCGB3A2 has any other roles, particularly in lung homeostasis and disease has not been demonstrated *in vivo*. The aim of this study was to address these questions in mice.

**Methods:**

A transgenic mouse line that expresses SCGB3A2 in the lung using the human surfactant protein-C promoter was established. Detailed histological, immunohistochemical, physiological, and molecular characterization of the *Scgb3a2*-transgenic mouse lungs were carried out. *Scgb3a2*-transgenic and wild-type mice were subjected to bleomycin-induced pulmonary fibrosis model, and their lungs and bronchoalveolar lavage fluids were collected at various time points during 9 weeks post-bleomycin treatment for further analysis.

**Results:**

Adult *Scgb3a2*-transgenic mouse lungs expressed approximately five-fold higher levels of SCGB3A2 protein in comparison to wild-type mice as determined by western blotting of lung tissues. Immunohistochemistry showed that expression was localized to alveolar type II cells in addition to airway epithelial cells, thus accurately reflecting the site of surfactant protein-C expression. *Scgb3a2*-transgenic mice showed normal lung development and histology, and no overt gross phenotypes. However, when subjected to a bleomycin-induced pulmonary fibrosis model, they initially exhibited exacerbated fibrosis at 3 weeks post-bleomycin administration that was more rapidly resolved by 6 weeks as compared with wild-type mice, as determined by lung histology, Masson Trichrome staining and hydroxyproline content, inflammatory cell numbers, expression of collagen genes, and proinflammatory cytokine levels. The decrease of fibrosis coincided with the increased expression of SCGB3A2 in *Scgb3a2*-transgenic lungs.

**Conclusions:**

These results demonstrate that SCGB3A2 is an anti-fibrotic agent, and suggest a possible therapeutic use of recombinant SCGB3A2 in the treatment of pulmonary fibrosis.

**Electronic supplementary material:**

The online version of this article (doi:10.1186/s12890-015-0065-4) contains supplementary material, which is available to authorized users.

## Background

Secretoglobin (SCGB) 3A2, also called UGRP1 (uteroglobin-related protein 1), is a member of the SCGB gene superfamily, consisting of cytokine-like secretory proteins of small molecular weight (~10 kDa) [1–3]. SCGB3A2 is highly expressed in airway Club cells, while low expression is observed at the growing tips of bronchi around embryonic day (E) 11.5 of mouse gestation [4]. SCGB3A2 plays a role in lung development as demonstrated using *ex vivo* embryonic lung organ cultures in the presence of SCGB3A2, and *in vivo* by administration of SCGB3A2 to pregnant female mice, followed by examination of pre-term pups [4]. Recently, SCGB3A2 was shown to be an early marker for Club cells (formerly called Clara cells) in conjunction with Notch signaling. Expression of SCGB3A2 appears earlier than SCGB1A1, the founding member of the SCGB gene superfamily, also called Club cell secretory protein (CCSP) or Club cell 10 kDa protein (CC10). SCGB1A1 was previously thought to be the only definitive marker for Club cells [5].

SCGB1A1 is the most well-characterized SCGB protein, exhibiting anti-inflammatory, anti-fibrotic, and immunomodulatory functions [6–9]. SCGB1A1 possesses phospholipase A_2_ (PLA_2_) inhibitory activity, which was thought to be at least partially responsible for the anti-inflammatory and immunomodulatory activity of SCGB1A1 [10, 11]. SCGB1A1 also exhibits tumor suppressor activity as demonstrated by decreased invasiveness of human lung adenocarcinoma-derived A549 cells *in vitro* [12], and the increased incidence of tumors in chemical carcinogenesis bioassay and the increased lung metastasis of B16F10 melanoma cells using *Scgb1a1*-null mice *in vivo* [13, 14].

SCGB3A2 also exhibits anti-inflammatory and anti-fibrotic activities [15, 16]; the anti-inflammatory function was originally suggested by the fact that *Scgb3a2* mRNA levels were reduced in the lungs of fungal-induced allergic inflammation model mice, which was almost restored by dexamethasone treatment [3]. Further, in the ovalbumin (OVA)-induced airway inflammation model mice, reduced levels of lung *Scgb3a2* mRNA were inversely correlated with the increased levels of proinflammatory cytokines, IL-5 and IL-9 in bronchoalveolar lavage fluid (BALF) [17, 18]. When OVA-induced airway inflammation model mice were intranasally administered recombinant adenovirus expressing SCGB3A2 before OVA challenge, OVA-induced airway inflammation was suppressed [15]. Lastly, *Scgb3a2*-null mice when subjected to OVA-inflammation model, showed exacerbated airway inflammation [19]. On the other hand, anti-fibrotic activity of SCGB3A2 was demonstrated by using a bleomycin (BLM)-induced mouse pulmonary fibrosis model [16]. This activity was through SCGB3A2-induced STAT1 phosphorylation and increased expression of inhibitory SMAD7, which inhibited the TGFβ signaling, resulting in reduced expression of various collagen genes and development of fibrosis [16] SCGB3A2 can also be used as a marker for pulmonary carcinomas in mice and humans [20, 21]. Taken together, SCGB3A2 has multiple biological activities, playing a role in lung homeostasis and function, and influencing various lung diseases. Whether SCGB3A2 possesses any other activities and the mechanisms for these activities have yet to be determined.

To understand the role of SCGB3A2 in lung homeostasis and diseases, an *Scgb3a2*-transgenic mouse was established that over-expresses SCGB3A2 in a lung-specific fashion under the control of the human surfactant protein C (SP-C) gene promoter. Detailed characterization demonstrated that the lungs of *Scgb3a2*-transgenic mice were histologically and functionally normal as compared to wild-type. When subjected to the BLM-induced pulmonary fibrosis model, however, they exhibited increased fibrosis at 3 weeks post-BLM administration, which was more quickly resolved by 6 weeks as compared to wild-type mice. These results demonstrate that SCGB3A2 has anti-fibrotic activity and suggest a potential use of SCGB3A2 as a therapeutic agent in treating lung fibrosis.

## Methods

### Transgenic construct

An expression plasmid with the human SP-C gene promoter (3.7 kb) cloned into pUC18 vector with SV40 small T intron and poly A (0.4 kb) (SPC3.7-SV40-pUC18), was provided from Dr. Jeffrey Whitsett (University of Cincinnati, OH) [22]. The mouse *Scgb3a2* cDNA that covers the entire protein coding sequence (50–427) was inserted into the SPC3.7-SV40-pUC18 plasmid. The resultant SPC3.7-SCGB3A2-SV40-pUC18 was double-digested with restriction enzymes, *Nde* I and *Not* I. The linearized SPC3.7-SCGB3A2-SV40 fragment was purified before microinjection into pronuclei of C57BL/6NCr mouse eggs. Production of *Scgb3a2*-transgenic mouse lines was confirmed by Southern blotting of genomic DNAs isolated from clipped mouse-tails.

### Northern blotting

Total RNA (3 μg) isolated from adult lungs of wild type and *Scgb3a2*-transgenic mice was electrophoresed on 1 % agarose gel containing 0.22 M formaldehyde and transferred onto nitrocellulose membrane (Immobilon-Ny+, Millipore, Billerica, MA). Filters were hybridized with SCGB3A2 probe obtained from *Eco* RI digestion of the SCGB3A2/pCR2.1 construct. Hybridization was performed in Perfect Hybridization solution (GE Healthcare Life Sciences, Piscataway, NJ) at 68 °C overnight. The membrane was washed twice with 2 x SSC containing 0.1 % SDS at 68 °C for 30 min, followed by exposure to a phosphoimager screen (Storm 840, GE Healthcare Life Sciences, Piscataway, NJ). Data processing was carried out using ImageQuant TL 2005 software (GE Healthcare Life Sciences).

### Western blotting

Lung from wild type and transgenic mice were frozen and crushed in 50 mM Tris-HCl, pH 8.0, 5 mM EDTA, 1 mM DTT, 1 mM phenylmethylsulfonyl fluoride (PMSF) with protein inhibitor cocktail (Roche Applied Science, Branford, CT). Protein concentrations were determined by Bradford assay (Bio-Rad Laboratories, Hercules, CA) with bovine serum albumin (BSA) as standard, and samples were mixed with equal volume of 2 x SDS sample buffer (125 mM Tris-HCl, pH 6.8, 4 % SDS, 20 % glycerol, 0.1 % mercaptoethanol). Ten microgram of sample was applied in each well of 20 % polyacrylamide gel and was run with running buffer of 50 mM Tris, 384 mM glycine, 2 % SDS. After electrophoresis, protein was transferred to Polyvinylidene fluoride (PVDF) membrane using a tank transfer system (Mini Trans-Blot Cell, Bio-Rad) with blotting buffer (50 mM Tris, 40 mM glycine, 20 % methanol) and electric field of 30 V for 6 h. To visualize SCGB3A2 band, PVDF membrane was treated as follows; 1 h blocking with PBST (phosphate buffered saline + 0.05 % Tween 20) +5 % BSA, 3 h incubation with 0.2 μg/ml polyclonal rabbit anti-SCGB3A2 IgG in PBST + 5 % BSA, PBST wash 3 times, incubation with 0.1 μl/ml horseradish peroxidase (HRP)-linked anti-Rabbit IgG F(ab’) fragment (GE Healthcare, NA9340) in PBST + 5 % BSA, and PBST wash 3 times before ECL plus (PerkinElmer, Waltham, MA) reaction. Polyclonal rabbit anti-SCGB3A2 antibody was produced as previously described [3]. The anti-SCGB3A2 IgG was purified using the Montage antibody purification kit (EMD Millipore, Billerica, MA) and used for all experiments. Labeled proteins were visualized using a SuperSignal West Pico Substrate (Thermo Scientific, Rockford, IL), and signals were detected using FluoChem HD2 System (ProteinSimple, San Jose, CA).

### Animal studies

All animal studies were carried out after approval by the National Cancer Institute Animal Care and Use Committee. Mouse embryonic lungs were collected from wild-type and *Scgb3a2*-transgenic pregnant females at various embryonic days (E). Noon of the day on which a vaginal plug was found was considered as E 0.5. Branching degree of *ex vivo* cultured embryonic lungs were counted after 3 days of culture as previously described [4]. Breathing score assessment was performed as previously described [4] according to the criteria described by Ozdemir et al. [23]; 0, no breathing; 1, gasping; 2, gasping/labored breathing; 3, labored breathing; 4, labored breathing/unlabored breathing; 5, unlabored breathing. For the BLM-induced pulmonary fibrosis model, mice of approximately 8 weeks old (at least 5 mice per group) were intratracheally intubated and dosed with BLM (1.2 U/kg) at day 0. Mice were killed on 3, 6, and 9 weeks after BLM intubation, and bronchoalveolar lavage (BAL) fluids obtained by lavaging lungs with 1 mL PBS [16]. The collected BAL fluids were used for counting and differentiating inflammatory cell numbers with Cytospin 4 (Thermo Scientific). PBS-treated mice killed at 3 weeks were used as normal control. Experiments were repeated more than 2 times, and the combined data points were used for analysis.

### ELISA

SCGB3A2 protein levels were determined by ELISA as previously described [24]. Briefly, samples diluted with a coating solution (500 mM bicarbonate buffer, pH 9.6), were applied onto each well of 96-well plates and the plates were incubated at 4 °C overnight. Calibration curves were constructed with twelve points by serially diluting a solution of recombinant mouse SCGB3A2 (1 μg/ml). The plates were washed four times with washing solution (PBS, pH 7.4 containing 0.5 % of Tween), followed by addition of blocking buffer (PBS, pH 7.4 containing 1 % of BSA) to each well. After incubation for 2 h at 37 °C, the plates were washed four times with the washing solution. Purified anti-mouse SCGB3A2 IgG [3] was applied to each well and the plates were incubated for 4 h at 37 or 4 °C overnight. The plates were washed seven times with the washing buffer. One hundred μl of ECL anti-rabbit IgG HRP-linked F(ab’) fragment (from donkey) was added to each well and the plates were placed at 37 °C for 2 h. After further washing, the amount of SCGB3A2 was determined by addition of 3,3′,5,5′-tetramethylbenzidine (TMB, Sigma) and was read at 450 nm after stopping the reaction by adding 1 N HCl.

### Lung histological analysis

Lungs were fixed in 4 % paraformaldehyde for one day at 4 °C, dehydrated, and embedded in paraffin. Lung tissues were sectioned at 4 μm and stained with hematoxyline and eosin (H&E). For immunohistochemistry, sections were at first rinsed with 0.05 % Triton-X 100 in PBS, and non-specific binding sites were blocked using 10 % normal goat serum in PBS containing 0.05 % Tween 20. Epitope retrieval was carried out using autoclave (5 min in citrate buffer, pH 6.0 or 1X TE, pH 9.0). After cooling to room temperature, the sections were incubated overnight at 4 °C with rabbit polyclonal anti-mouse SCGB3A2 [3] or anti-pro-surfactant protein-C (Seven Hills Bioreagents, Cincinnati, OH) primary antibodies. The sections were rinsed in distilled water, followed by treating with HRP-conjugated goat anti-rabbit IgG using the ABC method with a commercially available kit (Vector Laboratories, Burlingame, CA) according to the manufacturer’s instruction. Immunovisualization was carried out with 3, 3′-diaminobenzidine as substrate (Sigma, St Louis, MO), and counterstained with hematoxylin.

For double immunofluorescence labeling of adult lungs using two primary antibodies from the same species, the sections were first incubated with anti-SCGB3A2 antibody (1:1000) at 4 °C overnight, followed by labeled goat anti-rabbit IgG (Alexa Fluor 594 or 488, 1:200, Life Technology) as the secondary antibody for 1 h at room temperature. The sections were then incubated with 5 % rabbit serum for 1 h at room temperature, followed by incubation with unconjugated Fab Fragment goat anti-rabbit IgG for 1 h at room temperature. The sections were finally incubated with the secondary primary antibody, rabbit anti-pro-SP-C antibody (1:500), followed by labeled goat anti-rabbit IgG (Alexa Fluor 488 or 594, 1:200, Life Technology). Cell nuclei were identified by counterstaining with 4,6-diamino-2-phenylindolyl-dihydrochloride (DAPI, Life Technology). Fluorescence images were obtained and processed using a Zeiss 780 laser-scanning confocal microscope. Matching confocal planes were analyzed in all co-localization studies.

Severity of fibrosis was quantified from H&E stained entire lungs using the Ashcroft scoring system [25]. The degree of fibrosis was graded from 0 (normal lung) to 8 (severe distortion of structure, large fibrous areas, and honeycomb lesions). The mean score from all fields (magnification X200, average 30 fields/animal) was taken as the fibrosis score.

### Quantitation of hydroxyproline content in lung

Hydroxyproline content was measured using hydroxyproline assay kit from Biovision (Milpitas, CA) according to the manufacture’s instruction with slight modification. In brief, whole lungs were homogenized in dH_2_O, using 100 μl H_2_O for every 10 mg of tissue. To 100 μl of tissue homogenate, 200 μl concentrated HCl (6 N) was added in a pressure-tight, teflon capped vial, and the mixture was hydrolyzed at 120 °C for 3 h, followed by filtration through a 45 μm syringe filter (Millipore, Bedford, MA). Ten μl of hydrolyzed sample was transferred to a 96-well plate and was evaporated to dryness under vacuum, to which 100 μl Chloramine T reagent was added per well. After incubation at room temperature for 5 min, 100 μl p-dimethylaminobenzaldehyde reagent was added to each well and further incubated for 90 min at 60 °C. Absorbance was measured at 560 nm in a microplate reader (SpectroMax Plus384, Molecular Devices, Sunnyvale, CA).

### Microarray analysis

Lung total cellular RNAs were individually isolated from four mice each of no-treatment wild-type and *Scgb3a2* transgenic mice (approximately 8-weeks old) using RNeasy Mini Kit (Qiagen Science, Maryland, USA). The sample RNAs were labeled with Cy5, while pooled RNAs containing the same amount of RNA from each sample were labeled with Cy3 and used as a reference. Labeling was carried out using CyDye™ Post-Labeling Reactive Dye Pack (GE Healthcare) according to the manufacturer’s instructions. The purified Dye-coupled RNA samples were hybridized to an Agilent Whole Mouse Genome 4X44K oligo microarray kit (Agilent Techonologies, G4122F, Santa Club, CA), and were incubated for 17 h at 65 °C. The slides were washed, dried, and scanned using Agilent G26000 microarray scanner. The data were processed and analyzed by Genespring GX 11.0.2 software package (Agilent Technologies). All effective genes of microarray analysis were submitted to the Gene Expression Omnibus (GEO: ID # GSE47931).

### Quantitative RT-PCR analysis

Total RNAs isolated using TRIzol (Lifetechnologies) and digested with DNase I were reverse-transcribed by Superscript II reverse transcriptase (Life Technologies). Quantitative RT-PCR (qRT-PCR) was performed with ABI Prism 7900 Sequence Detection System (Applied Biosystems, Foster City, CA) using SYBR Green master mixture. The ΔΔ Ct method was used using β-actin or 18S as normalization control. PCR condition used was 50 °C, 2 min and 95 °C, 10 min followed by 95 °C, 15 s and 60 °C, 40 s for 40 cycles with the following primers: *Sftpa* (forward) 5′- ACT CCC ATT GTT TGC AGA ATC -3′, (reverse) 5′- AAG GGA GAG CCT GGA GAA AG -3′; *Sftpb* (forward) 5′- ACA GCC AGC ACA CCC TTG -3′, (reverse) 5′- TTC TCT GAG CAA CAG CTC CC -3′; *Sftpc* (forward) 5′- ATG AGA AGG CGT TTG AGG TG -3′, (reverse) 5′- AGC AAA GAG GTC CTG ATG GA -3′; *Sftpd* (forward) 5′- GAG AGC CCC ATA GGT CCT G -3′, (reverse) 5′- GTA GCC CAA CAG AGA ATG GC -3′; *Aqp1* (forward) 5′- TGC AGA GTG CCA ATG ATC TC -3′, (reverse) 5′- GGC ATC ACC TCC TCC CTA GT −3; *Col1a1* (forward) 5′-TAG GCC ATT GTG TAT GCA GC-3′, (reverse) 5′- ACA TGT TCA GCT TTG TGG ACC-3′; *Col3a1* (forward) 5′-TAG GAC TGA CCA AGG TGG CT-3′, (reverse) 5′- GGA ACC TGG TTT CTT CTC ACC-3′; *Col4a1* (forward) 5′-CAC ATT TTC CAC AGC CAG AG-3′, (reverse) 5′- GTC TGG CTT CTG CTG CTC TT-3′; *Col5a2* (forward) 5′-CAT GGA GAA GGT TTC CAA ATG-3′, (reverse) 5′- AAA GCC CAG GAA CAA GAG AA-3′; *Col12a1* (forward) 5′-TGA GGT CTG GGT AAA GGC AA-3′, (reverse) 5′- GTA TGA GGT CAC CGT CCA GG-3′; *Acta2* (forward) 5′-GTT CAG TGG TGC CTC TGT CA-3′, (reverse) 5′-ACT GGG ACG ACA TGG AAA AG-3′; *Ctgf* (forward) 5′-GCT TGG CGA TTT TAG GTG TC-3′, (reverse) 5′-CAG ACT GGA GAA GCA GAG CC-3′; *Mmp2* (forward) 5′-GGG GTC CAT TTT CTT CTT CA-3′, (reverse) 5′-CCA GCA AGT AGA TGC TGC CT-3′; *Mmp12* (forward) 5′-TTT GGA TTA TTG GAA TGC TGC-3′, (reverse) 5′-ATG AGG CAG AAA CGT GGA CT-3′; β-actin (forward) 5′-ATG GAG GGG AAT ACA GCC C-3′, (reverse) 5′-TTC TTT GCA GCT CCT TCG TT-3′; and 18S (forward) 5′- CGC GGT TCT ATT TTG TTG GT-3′, (reverse) 5′- AGT CGG CAT CGT TTA TGG TC-3′.

### Statistical analysis

All animal studies were carried out at least twice, each using at least 5–6 mice per group. Statistical analysis was carried out between wild-type and transgenic mice of various ages using a student’s *t*-test. *P* < 0.05 was considered significant.

## Results

### Production of *Scgb3a2*-transgenic mouse overexpressing SCGB3A2 in lung-specific fashion

**Fig. 1:**
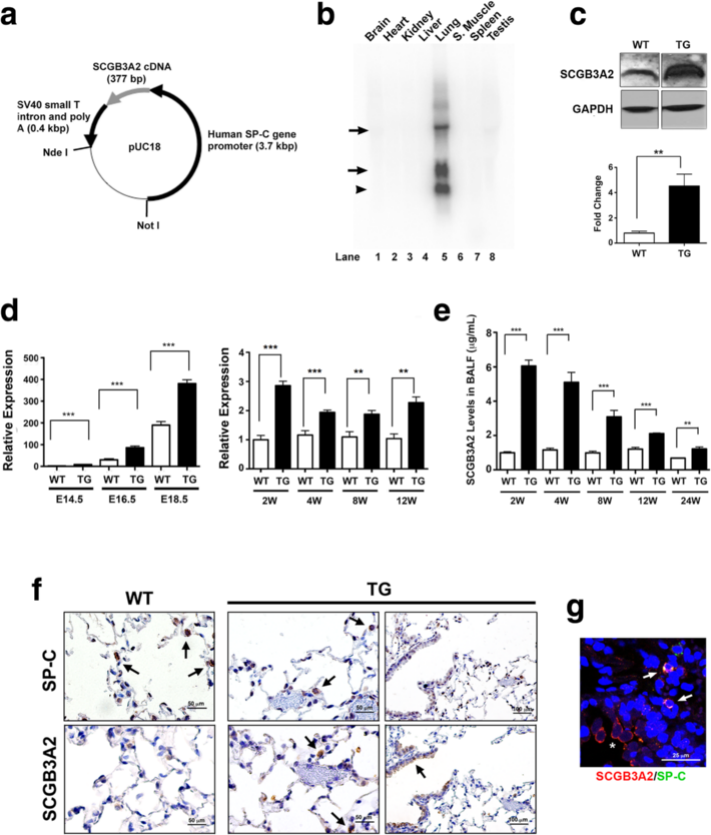
Generation of the SCGB3A2 transgenic mouse line. **a** Illustration of the construct showing the human SP-C gene promoter cloned into the pUC18 vector with SV40 small T intron and poly A. **b** Northern Blot analysis. The high level of *Scgb3a2* is observed only in lung tissue. The bands indicated by an arrow represent exogenously expressed *Scgb3a2* mRNAs. The highest mobility band shown by an arrowhead is derived from endogenous *Scgb3a2*. **c** Representative Western blot result showing increased level of SCGB3A2 protein in *Scgb3a2*-transgenic mouse lungs as compared with wild-type mice. GAPDH was used as a loading control. Lower panel shows the result of quantitation, normalized to GAPDH. *N* = 3. **d** qRT-PCR analysis of relative expression levels of *Scgb3a2* in lungs of various gestational days and ages of wild-type (WT) and transgenic (TG) mice. **e** SCGB3A2 levels in BALF of different ages of WT and TG mice. For D, E: *N* = 5 in each group. The results are shown as the mean ± SD. **P < 0.01, ***P < 0.001 by student *t*-test in comparison between WT and TG. **f** Immunohistochemistry for SP-C and SCGB3A2 in 4-month-old WT and TG mouse lungs. Arrows indicate representative positive signals in brown. TG lungs express SCGB3A2 in alveolar type II cells (middle panel) in addition to airway epithelial cells (right panel). Scales are as indicated. **g** Co-immunofluorescence analysis of TG mouse lungs for SP-C and SCGB3A2. Airway cells express only SCGB3A2 (red, shown by an asterisk) while alveolar type II cells express both SP-C and SCGB3A2 (yellow, shown by arrows). Scales are as indicated

Lung-specific over-expression of SCGB3A2 was achieved by producing a transgenic mouse line which harbors a transgenic construct containing a mouse *Scgb3a2* cDNA coding sequence, under control of the human SP-C gene promoter at the 5′ end, and the SV40 small T intron and poly A addition site at the 3′ end [22] (Fig. 1a). One founder mouse had high lung-specific expression of *Scgb3a2* as determined by Northern blotting (Fig. 1b). The highest mobility band corresponded to endogenously expressed *Scgb3a2*, while the transgene produced two bands with slower mobility.

Western blotting using a whole lung of 3-month-old mice demonstrated that the expression of SCGB3A2 protein was approximately five-fold higher in transgenic mouse lungs compared to wild-type control lungs (Fig. 1c). The mRNA level of *Scgb3a2* was measured by qRT-PCR using lung tissues and the protein level by bronchoalveolar lavage (BAL) fluids at various ages from embryonic day (E) 14.5 to 12 or 24-weeks old (Fig. 1d, e). The *Scgb3a2* mRNA levels drastically increased towards the end of gestation [26], and the levels in E18.5 transgenic embryo lungs were approximately two-fold higher than that of wild-type control lungs, which remained more or less at similar levels thereafter through 12 weeks (Fig. 1d). Transgenic mouse BAL fluids had about 6-fold higher levels of SCGB3A2 as compared to wild-type mice when measured at 2-weeks old (~6 μg/ml vs. ~1 μg/ml, respectively), which gradually decreased as the mice aged (Fig. 1e). Wild-type mice had ~1 μg/ml of SCGB3A2 for the entire period of 2 to 24 weeks. Immunohistochemistry and immunofluorescence analysis demonstrated that the transgenic mice expressed SCGB3A2 in alveolar type II cells, in addition to bronchiolar epithelial cells, the normal expression site for SCGB3A2 [3, 26, 27] (Fig. 1f, g), consistent with the expected expression of the alveolar type II cell-specific human SP-C [28]. Even though with this higher ectopic expression of SCGB3A2, both lungs of wild-type and transgenic mice looked histologically normal at 2 to 24-weeks old age. Further, scanning electron microscopy, using adult wild-type and *Scgb3a2*-null mouse lungs, demonstrated that ciliated cells appeared to be similar in morphology, size and number between wild-type and *Scgb3a2*-null airways (Additional file 1: Figure S1).

### Characterization of *Scgb3a2*-transgenic mouse lungs

Previous results showed that SCGB3A2 promoted lung development as demonstrated by branching morphogenesis in *ex vivo* organ culture studies as well as by *in vivo* administration of SCGB3A2 to pregnant females, followed by examination of preterm pups [4]. When E14.5, 16.5, and 18.5 wild-type and transgenic embryos were analyzed for body weight, body lengths, lung weights, and breathing scores of premature pups (Additional file 1: Figure S2A-D), no statistically significant differences were obtained between the two genotypes for all parameters examined. The development of embryonic lungs was also examined histologically, by immunohistochemistry and immunofluorescence. Like adults, E14.5 and 18.5 wild-type and transgenic embryo lungs presented similar histology (Additional file 1: Figure S2E). Immunohistochemical staining for SCGB3A2 confirmed that in E18.5 transgenic lungs, SCGB3A2 was strongly expressed in bronchiolar epithelial cells, the normal expression site for SCGB3A2 and the precursor to type II cells, the site of SP-C expression (Additional file 1: Figure S2E, bottom panel) [3, 26, 27]. Immunofluorescence studies using SCGB1A1 (CCSP) as a marker for Club cells, β-tubulin as a marker for ciliated cells, PGP9.5 as a maker for neuroendocrine cells, T1α as a marker for alveolar type I cells, and SP-C as a marker for alveolar type II cells showed no differences in the spatial and temporal expression patterns of these proteins between wild-type and transgenic embryo lungs at E14.5, 16.5 and 18.5 (Additional file 1: Figure S2F, and data from E14.5 and 16.5 not shown). These results thus suggested that both wild-type and transgenic embryo lungs developed similarly during lung morphogenesis.

In order to examine whether *Scgb3a2*-transgenic mice have normal lung function, lung chord lengths and lung volumes were measured to calculate surface areas using the point and intersection counting method [29]. The surface areas were not different between wild-type and transgenic mice of either 2-, 3-, 4-, or 12-weeks-old (Additional file 1: Figure S3A). Further, when mice were subjected to MouseOx to monitor O_2_ saturation, heart rate, pulse distention, and breath rate, none of these parameters had significant differences between wild-type and transgenic mice of either ages (Additional file 1: Figure S3B-E). These results suggested that *Scgb3a2*-transgenic mice had normal lung function at two weeks and up to 12 weeks old.

### Accelerated resolution of BLM-induced pulmonary fibrosis in *Scgb3a2*-transgenic lungs

**Fig. 2:**
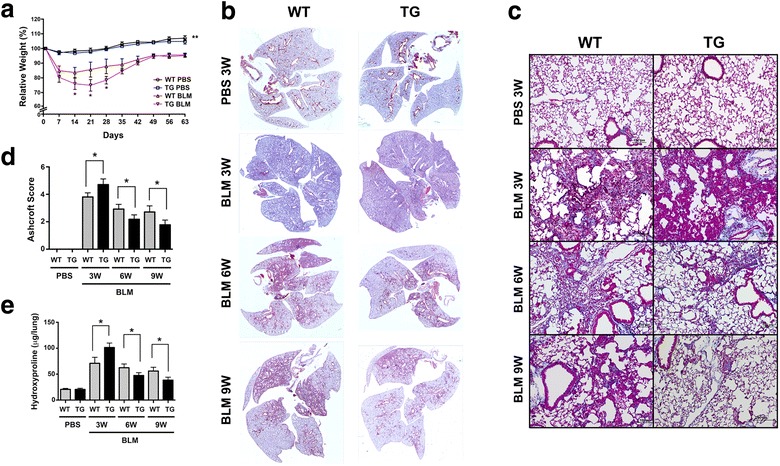
BLM-induced pulmonary fibrosis in wild-type (WT) and *Scgb3a2*-transgenic (TG) mice. **a** Body weight curves of mice intubated and treated with BLM on day 0, followed by necropsy on day 63. Body weights are shown as the percentage of Day 0 weight set as 100 %. *P < 0.05, WT-BLM group vs. TG-BLM, **P < 0.01, BLM-treated groups vs. PBS-treated groups. **b** Whole lung H&E images of WT and TG lungs at 3 weeks (BLM 3 W), 6 weeks (BLM 6 W), and 9 weeks (BLM 9 W) post-BLM treatment, and their corresponding PBS-treated lungs collected at 3 W (PBS) as control. Magnification 40X images were stitched. **c** Masson Trichrome staining of lung sections of WT and TG mice at 3, 6, and 9 weeks post-BLM treatment. Magnification, 100X. **d** Ashcroft scores of BLM-induced damaged areas at 3, 6, and 9 weeks post-BLM treatment. PBS control levels are those from 3 W. **e** Hydroxyproline content at 3, 6, and 9 weeks post-BLM treatment. PBS control levels are those from 3 W. *N* > 6 in each group. The results are shown as the mean ± SD. *P < 0.05, NS, not significant. Statistical analysis was carried out by using student’s *t*-test

In order to examine whether *Scgb3a2*-transgenic mice exhibit any phenotypes upon challenge, both wild-type and *Scgb3a2*-transgenic mice were subjected to the BLM-induced pulmonary fibrosis model. After BLM intubation, both wild-type and transgenic mice lost weight at similar rates for the first five days, from which transgenic mice lost significantly more weight as compared to wild-type (Fig. 2a). On day 21 (3 weeks post-BLM), wild-type and transgenic mice weighed 85 and 75 % of their original weight, respectively. However, after day 21, the weight of transgenic mice gradually increased and reached the wild-type weight by day 49. There was no difference in the survival curves between BLM-treated wild-type and *Scgb3a2*-transgenic mice (Additional file 1: Figure S4). Histological examination revealed that both BLM-treated wild-type and *Scgb3a2*-transgenic mice developed extensive pulmonary fibrosis by 3 weeks based on a whole lung H&E stained images (Fig. 2b) and Masson Trichrome staining (Fig. 2c). When the extent of fibrous damage was calculated using these histological sections with the Ashcroft score system, BLM-treated *Scgb3a2*-transgenic mice had slightly increased damaged areas by 3 weeks as compared to the BLM-treated wild-type group with statistical significance (Fig. 2d). Ashcroft scores were then decreased by 6 weeks in both wild-type and transgenic mice, which continued until 9 weeks post-BLM treatment. BLM-induced pulmonary fibrosis with single dose administration is known to partially resolve after weeks of BLM treatment due to unknown reasons [30–32]. At both 6 and 9 weeks post-BLM administration, the Ashcroft score was significantly lower in transgenic lungs than their wild-type counterparts, suggesting that BLM-induced pulmonary fibrosis may have dissolved more quickly in transgenic lungs than wild-type lungs. This result was supported by Masson Trichrome staining that detects collagen fibers (Fig. 2C), and hydroxyproline contents (Fig. 2e), both of which were increased in both transgenic and wild-type lungs at 3 weeks. These levels gradually decreased post-BLM over the course of 9 weeks. The transgenic lungs demonstrated a much more rapid decrease of hydroxyproline content; by 9 weeks post-BLM, the level in the transgenic lungs was less than a half of its peak level, and was only 2 fold higher than the control level.

### Gene expression analysis of BLM-induced pulmonary fibrosis in *Scgb3a2*-transgenic lungs

**Fig. 3:**
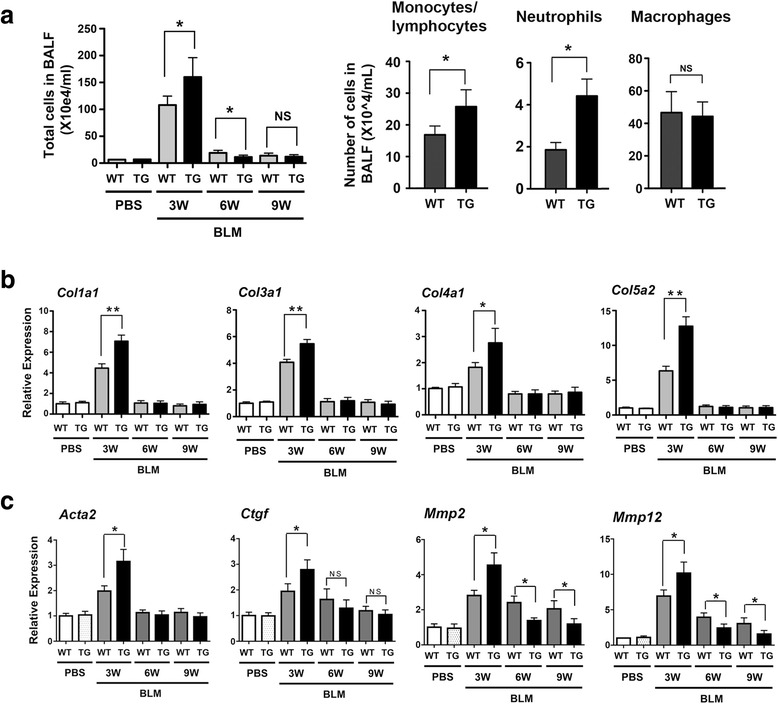
Characterization of BLM-induced pulmonary fibrosis-harboring lungs of wild-type (WT) and *Scgb3a2*-transgenic (TG) mice. **a** Number of inflammatory cells in BAL fluids at 3, 6, and 9 weeks post-BLM treatment and PBS control groups collected at 3 weeks. (right panel) Number of monocytes/lymphocytes, neutrophils, and macrophages from 3 weeks post-BLM treatment mice were separately counted. *P < 0.05 by student’s *t*-test. **b** qRT-PCR analysis for various mRNA levels. The results are shown as the mean ± SD from *N* = 7-10. *P < 0.05, **P < 0.01, NS, not significant by student’s *t*-test

Total inflammatory cell numbers in BALF and levels of mRNAs encoding collagen 1a1, 3a1, 4a1, and 5a2 were drastically increased at 3 weeks post-BLM treatment and the levels were higher in *Scgb3a2*-transgenic than wild-type lungs (Fig. 3a left panel, b). Collagen 12a1 mRNA levels did not change after BLM treatment in both mouse lines (data not shown). The increased inflammatory cells were due to increased numbers of monocytes/lymphocytes and neutrophils (Fig. 3a right panel). The number of macrophages was not different between *Scgb3a2*-transgenic and wild-type lungs. Inflammatory cell numbers and all collagen mRNA levels rapidly decreased to almost control levels in both BLM-treated wild-type as well as *Scgb3a2*-transgenic mouse lungs by 6 weeks post-BLM treatment. The expression of mRNAs encoded by other genes involved in bleomycin-induced fibrosis such as *Acta2*, *Ctgf*, *Mmp2*, and *Mmp12* were also determined (Fig. 3c). The expression of all these mRNAs markedly increased at 3 weeks post-BLM with the levels in transgenic lungs being higher than those of wild-type lungs. However, the increased expression again quickly decreased by 6 weeks post-BLM; in particularly expression in the transgenic lungs returned to control levels.

**Fig. 4:**
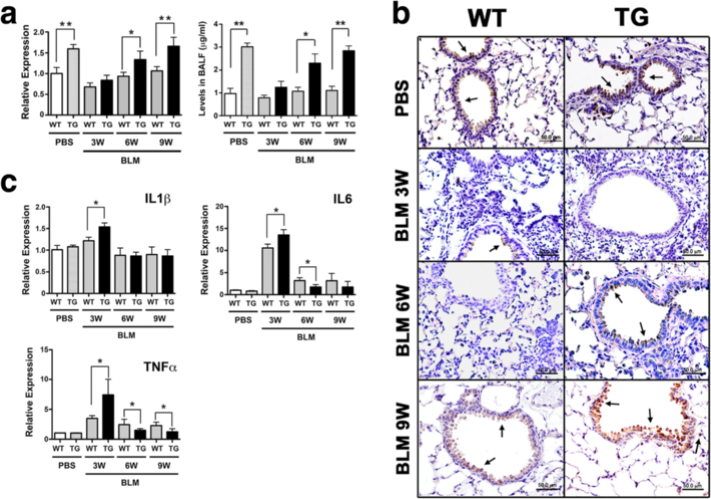
SCGB3A2 and proinflammatory cytokine levels in lungs of wild-type (WT) and *Scgb3a2*-transgenic (TG) mice. **a** Levels of lung *Scgb3a2* mRNAs (left panel) and SCGB3A2 proteins in BALF (right panel) in WT and TG mice at 3, 6, and 9 weeks post-BLM treatment and PBS collected at 3 weeks as control. **b** Representative immunohistochemistry of SCGB3A2 in WT and TG mice at 3 and 6 weeks post-BLM treatment and PBS collected at 3 weeks as control. Arrow indicates representative SCGB3A2 positive signals found in the airway epithelial cells. Scale bar: 50 μm. **c** qRT-PCR analysis of lung mRNAs for proinflammatory cytokines, IL-1β, IL-6, and TNFα at 3, 6, and 9 weeks post-BLM treatment and PBS as control. *N* > 6 in each group. *P < 0.05, **P < 0.01 by student’s *t*-test

The levels of *Scgb3a2* mRNA and protein in BALF were determined by qRT-PCR and ELISA using PBS and BLM-treated lungs and BALF of wild-type and transgenic mice, respectively (Fig. 4a). The differences in *Scgb3a2* mRNA and protein levels initially observed between *Scgb3a2*-transgenic and wild-type mice were no longer found in 3 week post-BLM-treated lungs, and their levels were similar to those of PBS treated wild-type lungs. *Scgb3a2* mRNA and protein levels of transgenic mice gradually returned to the PBS control levels by 6 weeks; the levels of transgenic mice again became higher than those of wild-type. Immunohistochemistry did not show clear positive staining for SCGB3A2 in 3 weeks-post-BLM lungs of both genotypes, however very weak signals were sometimes noted in wild-type bronchiolar epithelial cells (Fig. 4b). The expression of SCGB3A2 became detectable in the transgenic bronchiolar epithelial cells at 6 weeks post BLM-treatment while no expression was found in wild-type lungs. SP-C expression remained almost undetectable until 6 weeks after BLM treatment in both wild-type and *Scgb3a2*-null lungs (Additional file 1: Figure S5 upper panel). By 9 weeks post-BLM, the expression of SCGB3A2 was found in both wild-type and transgenic lungs, with the intensity apparently being stronger in the transgenic lungs. Interestingly, ectopic SP-C expression was found in the bronchiolar epithelial cells in addition to type II cells; some co-expressing with SCGB3A2 (Additional file 1: Figure S5 middle and lower panels). The ectopic expression of SP-C may contribute to the higher expression of SCGB3A2 in the epithelial cells at 9 weeks post-BLM.

Levels of mRNAs encoding inflammatory cytokines, IL1β, IL6, and TNFα were also determined by qRT-PCR (Fig. 4c). *Il6* and *Tnfa* mRNAs were significantly increased in 3 weeks post-BLM lungs of both *Scgb3a2* transgenic and wild-type mice, and the levels were higher in lungs of *Scgb3a2* transgenic than wild-type mice. The levels were then reduced close to those of the PBS group at 6 weeks post-BLM. The *Scgb3a2* transgenic mouse lungs had a much more drastic reduction in *Il6* and *Tnfa* mRNAs as compared to wild-type. These results altogether demonstrated that *Scgb3a2*-transgenic mice resolved BLM-induced pulmonary fibrosis more rapidly than PBS control.

### Microarray analysis of 3 weeks post-BLM *Scgb3a2*-transgenic mouse lungs

**Fig. 5:**
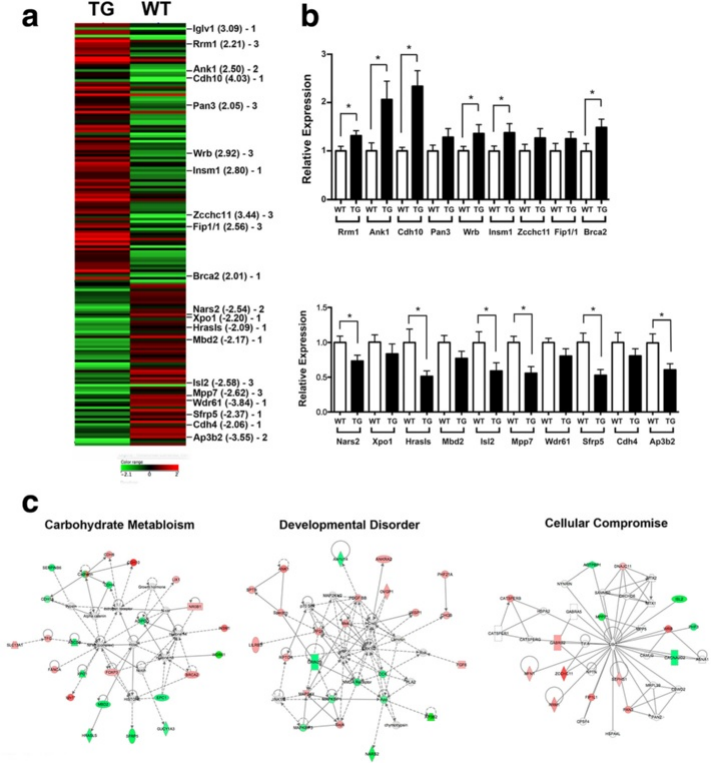
Microarray analysis. **a** Heat map diagram of differentially expressed genes in normal *Scgb3a2*-transgenic (TG) lungs vs. wild-type (WT) lungs. All genes >2 fold with *P* < 0.05 differences were used for the analysis. The average of 4 samples in each group is shown. Green and red indicate down- and up-regulation of gene expression, respectively. The scale is shown at the bottom. Genes found in top three pathways as shown in C are indicated with the fold differences in parenthesis. The number (1, 2, or 3) means the pathway to which indicated genes belong in C. **b** qRT-PCR analysis of highly up- or down-regulated genes indicated in A using RNAs isolated from untreated WT and TG mouse lungs. The results are shown as the mean ± SD. *N* > 6, *P < 0.05 by student’s *t*-test. **c** Top three pathways, Carbohydrate metabolism (pathway 1), Developmental disorder (pathway 2), and Cellular compromise pathways (pathway 3) determined by Ingenuity Pathway Analysis using genes identified by microarray analysis that have >1.5 fold with *P* < 0.05 differences

In order to obtain an insight into the slightly increased phenotype of 3 weeks post-BLM-treated *Scgb3a2*-transgenic mouse lungs as compared to wild-type mice, microarray analysis was carried out using RNAs isolated from lungs of wild-type and *Scgb3a2*-transgenic mice without any treatment. Two hundred and sixty-eight and 165 genes were respectively up and down-regulated more than 1.5 fold with *P* < 0.05 in *Scgb3a2*-transgenic mouse lungs as compared to wild-type lungs (Fig. 5a and Additional file 2: Table S1). Several genes with most differential expression were selected and were subjected to qRT-PCR analysis (Fig. 5b). All of the genes were up or down-regulated with statistical significance or showed a trend of up or down-regulation in lungs of *Scgb3a2*-transgenic mice as compared to wild-type mice as predicted from the array results. These genes were the main constituents of the top three pathways; Carbohydrate Metabolism, Developmental Disorder, and Cellular Compromise pathways as determined by the Ingenuity Pathway Analysis program (Fig. 5c). These results suggested that several metabolic and/or cellular systems were perturbed in the transgenic lungs. Further, Bio Functions analysis identified inflammation, cell dynamics, and/or cell trafficking-related functions as the Top Bio Functions (Table 1). These results suggested that the *Scgb3a2*-transgenic mouse lungs had altered cellular metabolism and homeostasis with higher expression levels of genes associated with inflammation, and cellular signaling and movement, which may have played a role in the more susceptible phenotype of *Scgb3a2*-transgenic mouse lungs to BLM-induced fibrosis at 3 weeks post-BLM treatment.

**Table 1:** Top Bio Functions identified in lungs of normal *Scgb3a2*-transgenic vs. wild-type mouse

Name of functions	*p*-value	# of molecules
Diseases and disorders		
Inflammatory response	6.76E-04–2.75E-02	9
Organismal injury and abnormalities	1.12E-03–4.06E-02	16
Neurological response	1.83E-03–4.10E-02	20
Nutritional disease	1.83E-03–1.83E-03	3
Cancer	2.76E-03–4.10E-02	15
Molecular and cellular functions		
Cellular development	8.79E-05–3.99E-02	25
Cell growth and proliferation	8.79E-05–3.52E-02	21
Drug metabolism	1.91E-04–2.75E-02	7
Small molecule biochemistry	5.68E-04–4.10E-02	20
Cellular movement	6.76E-04–3.68E-02	17
Physiological system development and function		
Cardiovascular system development and function	8.79E-05–2.80E-02	10
Connective tissue development and function	8.79E-05–4.10E-02	10
Hematological system development and function	6.76E-04–4.10E-02	14
Immune cell trafficking	6.76E-04–4.10E-02	8
Nervous system development and function	1.12E-03–4.10E-02	24

## Discussion

This study describes the establishment of an *Scgb3a2*-transgenic mouse line that expresses SCGB3A2 in a lung-specific fashion under regulation of the human SP-C gene promoter. Since SP-C is a marker for alveolar type II cells [28, 33], the promoter for this gene was used for transgenic over-expression of SCGB3A2 in the alveolar areas because in a previous study using the BLM-induced pulmonary fibrosis model, pulmonary fibrosis was reduced by intravenous administration of SCGB3A2 [16, 24]. In the latter case, it is likely that SCGB3A2, an airway-specific protein, reached the alveolar areas as well as airways through blood circulation at similar levels, resulting in the suppression of fibrosis. Thus, we hypothesized that this situation may be better obtained by the use of type II-specific over-expression of SCGB3A2. Further, chose not to use the tetracycline inducible system because it is not known whether and/or how doxycycline affects formation and/or resolution of BLM-induced pulmonary fibrosis, in an otherwise well-established BLM-induced pulmonary fibrosis model.

SCGB3A2 expression faithfully mimicked the expression pattern of the lung specific SP-C gene. Over-expression of SCGB3A2 in transgenic mouse lungs was confirmed by qRT-PCR for mRNA and Western blotting for protein using lung tissues, and levels in BALF by ELISA. The difference in mRNA expression levels between wild-type and transgenic mouse lungs was observed as early as E14.5. The SCGB3A2 mRNA and protein levels in lung were highest at around two weeks of age. Immunohistochemistry further demonstrated expression of SCGB3A2 in airway epithelial cells, the endogenous expression site for SCGB3A2 as well as type II cells, the specific site for SP-C expression [28, 33]. Since alveolar type II cells constitute ~15 % of the peripheral lung cells [34], the expression of SCGB3A2 in type II cells is likely to be a main contributor to over-expression of SCGB3A2 in the transgenic lung. Two different sizes of *Scgb3a2* mRNAs were observed by Northern blotting for *Scgb3a2*-transgenic mice that were larger than that of endogenously expressed *Scgb3a2*. We do not know the exact reason for this phenomenon. It could be due to longer polyA tails present in the transgene-derived mRNA. Alternatively, since the transgene construct contains a *Scgb3a2* cDNA that does not have an intron, the mRNA processing may have been affected, resulting in a larger mRNA. The way the transgene was inserted to a chromosome might also have affected the size of *Scgb3a2* mRNA.

While *Scgb3a2*-transgenic lungs exhibited no phenotypes with normal lung function, they showed exacerbated fibrosis at 3 weeks after subjected to the BLM-induced pulmonary fibrosis model as determined by lung histology, hydroxyproline content, inflammatory cell numbers, collagen gene expression, and inflammatory cytokine levels. Microarray analysis revealed that this was due to altered expression of genes caused by ectopic overexpression of SCGB3A2 that are involved in inflammation, cell dynamics, and/or cell trafficking-related functions as compared to wild-type. These changes in gene expression patterns may have affected metabolism and homeostasis of the transgenic lungs, which rendered the transgenic lungs more susceptible to BLM challenge. However the changes may be too subtle to affect lung homeostasis without challenge since we did not detect any gross metabolic or abnormal inflammatory phenotypes in the transgenic mice. Of most interest is that after 3 weeks, the fibrosis of *Scgb3a2*-transgenic lungs quickly resolved as compared with wild-type lungs. BLM-induced pulmonary fibrosis is known to partially resolve after weeks of BLM treatment due to unknown reasons [30–32]. Previously we demonstrated that SCGB3A2 exhibits anti-fibrotic activity [16, 24]. The anti-fibrotic activity of SCGB3A2 was due to suppression of the TGFβ-induced differentiation of fibroblasts to myofibroblasts, a hallmark of the fibrogenic process through increased phosphorylation of STAT1 and expression of SMAD7, and decreased phosphorylation of SMAD2 and SMAD3 [16]. The BLM-induced injury includes damage to and involvement of alveolar and bronchial epithelium, and decreased SCGB1A1 expression in the airway epithelial cells after BLM treatment was described following BLM [35]. In the current study, SCGB3A2 overexpression predisposed mice to a more severe phenotype 3 weeks post-BLM, at which time SCGB3A2 expression was almost at the level of wild-type mice, suggesting that changes in gene expression patterns in transgenic mice predominate in determination of the phenotype. However by 9 weeks post-BLM, the expression of SCGB3A2 in transgenic lungs recovered to levels found in pre-BLM lungs, which was significantly higher than that of wild-type lungs. Ectopic expression of SP-C was also observed in the bronchial epithelial cells, which may have contributed to the more rapid increase in SCGB3A2 expression in the transgenic lungs. Previously it was shown that after BLM administration, SP-C is co-expressed in bronchial epithelial cells with SCGB1A1, the marker for Club cells that is the site for SCGB3A2 expression [36, 37]. The temporary SP-C-positive, SCGB1A1-positive cells were suggested to eventually differentiate into type II cells during the repair after severe pulmonary injury [36, 37]. In the current study, the resolution of fibrosis coincided with the rapid increase of SCGB3A2 expression in lungs of *Scgb3a2*-transgenic mice, suggesting the anti-fibrotic activity of SCGB3A2. How SCGB3A2 promotes natural resolution of BLM-induced pulmonary fibrosis requires further studies.

In the single dose BLM model, pulmonary fibrosis develops through sequential events after BLM administration; first in inflammation phase (≤7 days), followed by fibrosis phase (≥7 days) [30]. Many compounds were reported as anti-fibrotic agents, however most of them (over 220 compounds) were given ≤7 days of BLM administration and thus considered as preventive agents. Only a handful compounds were administered in the fibrosis phase as therapeutic agents. The present studies demonstrated that expression of SCGB3A2 markedly increased in transgenic mice after the severity of fibrosis reached peak levels, and thus the situation may resemble that of SCGB3A2 being administered in the therapeutic phase, which likely resulted in the rapid decrease of fibrosis. These results are in good agreement with the previous reports using BLM-induced pulmonary fibrosis model mice with intravenously administered SCGB3A2 that SCGB3A2 possesses anti-fibrotic activity and may be used as a therapeutic agent in treatment of pulmonary fibrosis [16, 24].

## Conclusions

A transgenic mouse over-expressing SCGB3A2 in lung-specific fashion under the promoter of human SP-C gene was established. The lungs of the *Scgb3a2*-transgenic mice were histologically and functionally normal. When these mice were subjected to the BLM-induced pulmonary fibrosis model, a slightly exaggerated fibrosis was initially noted at 3 weeks post-BLM, however the fibrosis more rapidly resolved in *Scgb3a2*-transgenic mice as compared to wild-type by 6 weeks post-BLM. These results demonstrate the possible therapeutic use of SCGB3A2 in treatment of lung fibrosis.

## Additional files

Additional file 1: Figure S1. Scanning electron microscopy of wild-type (WT) and *Scgb3a2*-transgenic mouse (TG) airways. **Figure S2.** Characterization of *Scgb3a2*-transgenic embryo lungs. **Figure S3.** Lung morphometric analysis. **Figure S4.** Survival curve of wild-type (WT) and *Scgb3a2*-transgenic mice (TG) after BLM treatment. **Figure S5.** SP-C expression in wild-type (WT) and *Scgb3a2*-transgenic mouse (TG) lungs at 6 and 9 weeks post-BLM administration. Additional file 2: Table S1. Up and down-regulated genes in lungs of *Scgb3a2*-transgenic vs. wild-type mice.

## Abbreviations

SCGB: Secretoglobin
BLM: Bleomycin
