# Supplementary material for: Transgenically-expressed secretoglobin 3A2 accelerates resolution of bleomycin-induced pulmonary fibrosis in mice
Source: BMC Pulm Med. 2015 Jul 16;15:72. doi: 10.1186/s12890-015-0065-4 (PMC4504078; doi:10.1186/s12890-015-0065-4)

# Supporting materials

## Materials and Methods

### Immunofluorescence analysis of embryonic lungs

Immunofluorescence of embryonic lungs was carried out using the following antibodies; SCGB1A1 (CCSP) as a marker for Club cells (goat anti-CC10, Santa Cruz, 1:1500), β-tubulin as a marker for ciliated cells (mouse anti-βTubulinIV, Abcam, 1:300), PGP9.5 as a maker for neuroendocrine cells (rabbit anti-PGP9.5, Dako, 1:300), T1α as a marker for alveolar type I cells (hamster anti-T1α, DSHB, 1:1000), and SP-C as a marker for alveolar type II cells (rabbit anti-pro-SP-C, Abcam, 1:500) with secondary antibodies conjugated to Alexa Fluor 488, 568, 647 (Donkey, Invitrogen). Samples were imaged using a Zeiss LSM710 metaconfocal laser-scanning microscope.

### Lung functional analysis

Surface areas were calculated using lung volume and chord length determined using the method previously described [1]. Hemodynamic parameters such as oxygen saturation, heart rate, pulse distention, and breath rate were measured using MouseOx® small animal pulse oximeter (Starr Life Sciences, Oakmont, PA).

### Scanning Electron Microscopy (SEM)

SEM was carried out using a modified method of previous description [2, 3]. Briefly, the trachea and the bronchi were separated from the lung. The tissues were fixed by immersion in 1% glutaraldehyde for 1 h. The lumina were exposed by removing the

dorsal half of the airway by microdissection and fixed again for 2-3 h with 1% glutaraldehyde. The fixative was made up in 0.1 M phosphate buffer (pH 7.4). Fixation and washing were carried out on ice or at 4°C, the tissues were then dehydrated through a graded ethanol series and substituted with *tert*-butanol. The tissues were frozen at -20°C for a few minutes and then freeze-dried by JFD-320 freeze drying device (JEOL, Tokyo, Japan). The tissue specimens were mounted on brass studs with aluminum conducting tape and coated with a 20 nm layer of platinum in a JFC-1600 ion sputterer (JEOL, Tokyo, Japan). The specimens were examined with a JSM-6510LV SEM (JEOL) operated at 7 kV. All photographs were taken in the secondary electron mode with the beam incident to the surface of the issue.

## References


1. Knudsen L, Weibel ER, Gundersen HJ, Weinstein FV, Ochs M: **Assessment of air space size characteristics by intercept (chord) measurement: an accurate and efficient stereological approach**. *J Appl Physiol* 2010, **108**(2):412-421.
2. Abe H, Oikawa T: **Observations by scanning electron microscopy of oviductal epithelial cells from cows at follicular and luteal phases**. *Anat Rec* 1993, **235**(3):399-410.
3. Abe H, Onodera M, Sugawara S: **Scanning electron microscopy of goat oviductal epithelial cells at the follicular and luteal phases of the oestrus cycle**. *J Anat* 1993, **183 ( Pt 2)**:415-421.

## Figure legends

**Figure S1. Scanning electron microscopy of wild-type (WT) and *Scgb3a2*-transgenic mouse (TG) airways.** Magnification: X2000 **(**Upper panel) and X5000 (lower panel). Scale bars are as indicated.

**Figure S2. Characterization of *Scgb3a2*-transgenic embryo lungs. (A-D)** Body weight **(A),** body length **(B)**, lung weight **(C)** measurements, and breathing score assessment **(D)**. Embryonic Day (E) 14.5, 16.5 and 18.5 pups were removed from dams in wild-type (WT) and *Scgb3a2*-transgenic mice (TG), and were subjected to these analyses. N>7. A dot represents a lung or an embryo. The results show the mean ± S.D. No statistically significant differences were obtained in any parameters analyzed. **(E)** Histological characterization of *Scgb3a2*-transgenic mouse lungs at various embryonic stages. Upper and middle panels: representative H&E staining (H&E) of E14.5 and 18.5 wild-type (WT) and *Scgb3a2*-transgenic mouse (TG) lungs. Scale bar: 50 μm. Lower panel: representative immunostaining for SCGB3A2 (SCGB3A2) in E18.5 WT and TG embryonic lungs. Brown color indicates positive staining (representatives shown by an arrow). Scale bar: 20 μm. Insert in TG: Immunofluorescence results showing co-expression of SP-C and SCGB3A2 (in yellow due to mixture of red for SP-C and green for SCGB3A2, shown by arrowheads). N>3. **(F)** Immunofluorescence analysis of E18.5 embryo lungs. E18.5 wild-type (WT) and *Scgb3a2*-transgenic (TG) embryo lungs were subjected to immunofluorescence for SCGB1A1 (CCSP), β-tubulin, PGP9.5, T1α, and

SP-C. Representative results are shown from N=2. TB: terminal bronchiole. Scale bar: 20 μm.

**Figure S3. Lung morphometric analysis. (A)** Surface areas of wild-type (WT) and *Scgb3a2*-transgenic mouse (TG) lungs determined at various ages. **(B-E)** Hemodynamic parameters; Oxygen saturation **(B)**, heart rate **(C)**, pulse distention **(D)** and breath rate **(E)**, measured in different age WT and TG mice. N>3. Bar represents the mean ± S.D. No statistically significant differences were obtained in any parameters analyzed.

**Figure S4. Survival curve of wild-type (WT) and *Scgb3a2*-transgenic mice (TG) after BLM treatment.** No statistical difference was found in the survival curve of WT and TG mice. N>25 in each group.

**Figure S5. SP-C expression in wild-type (WT) and *Scgb3a2*-transgenic mouse (TG) lungs at 6 and 9 weeks post-BLM administration.** Upper, middle panels: Immunohistochemistry for SP-C of 6 weeks (upper) and 9 weeks (middle) post-BLM lungs. Expression was not observed at 6 weeks post-BLM, but the high expression was found in epithelial and type II cells in both wild-type and transgenic mouse lungs (representatives shown by arrows). Lowe panel: Immunofluorescence for SP-C (red) and SCGB3A2 (green) are shown. The bronchial epithelial cells express both proteins (yellow, representative shown by an arrow). Scale bar: 50 μm.

Figure S1

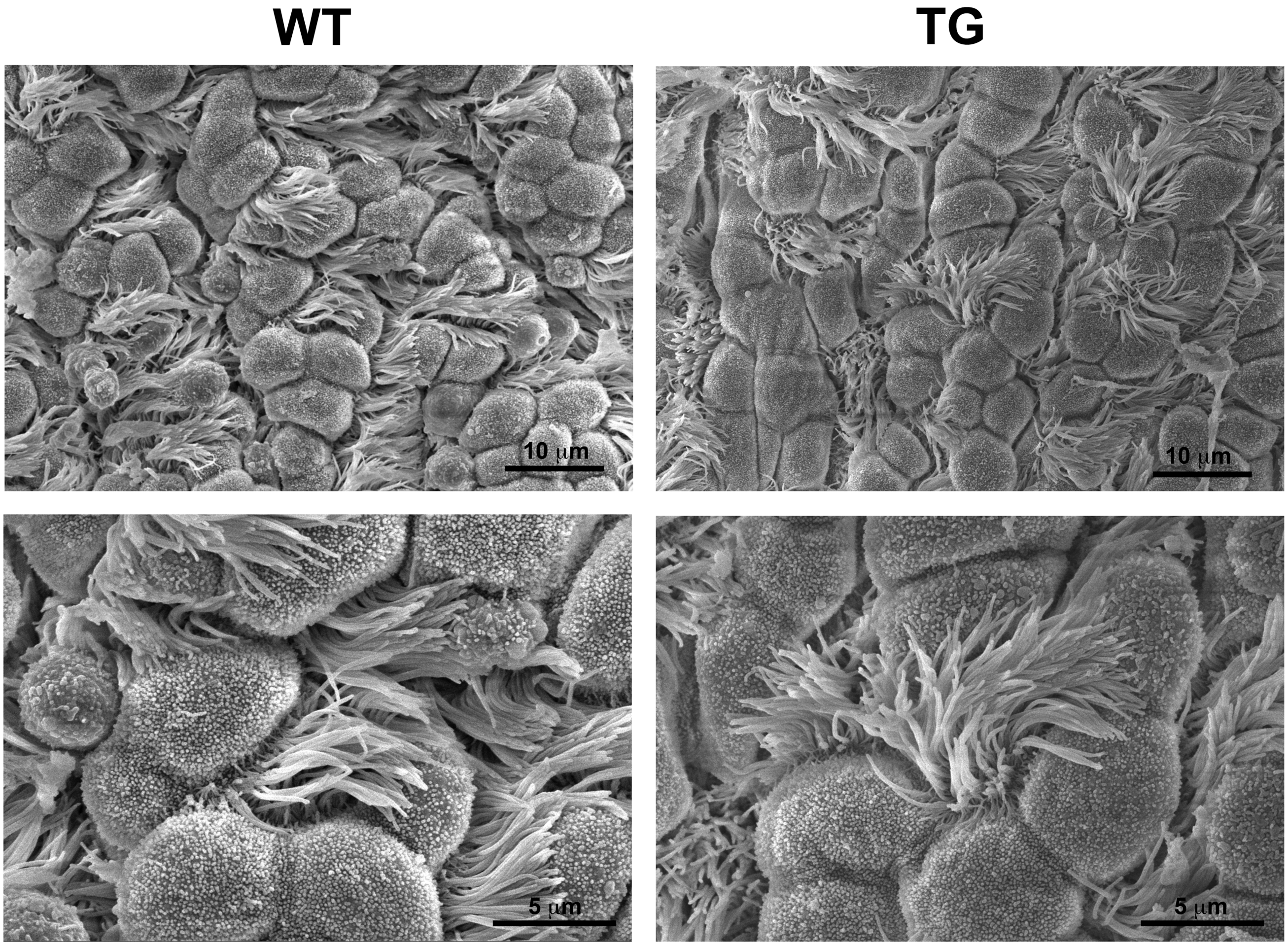

# Figure S2

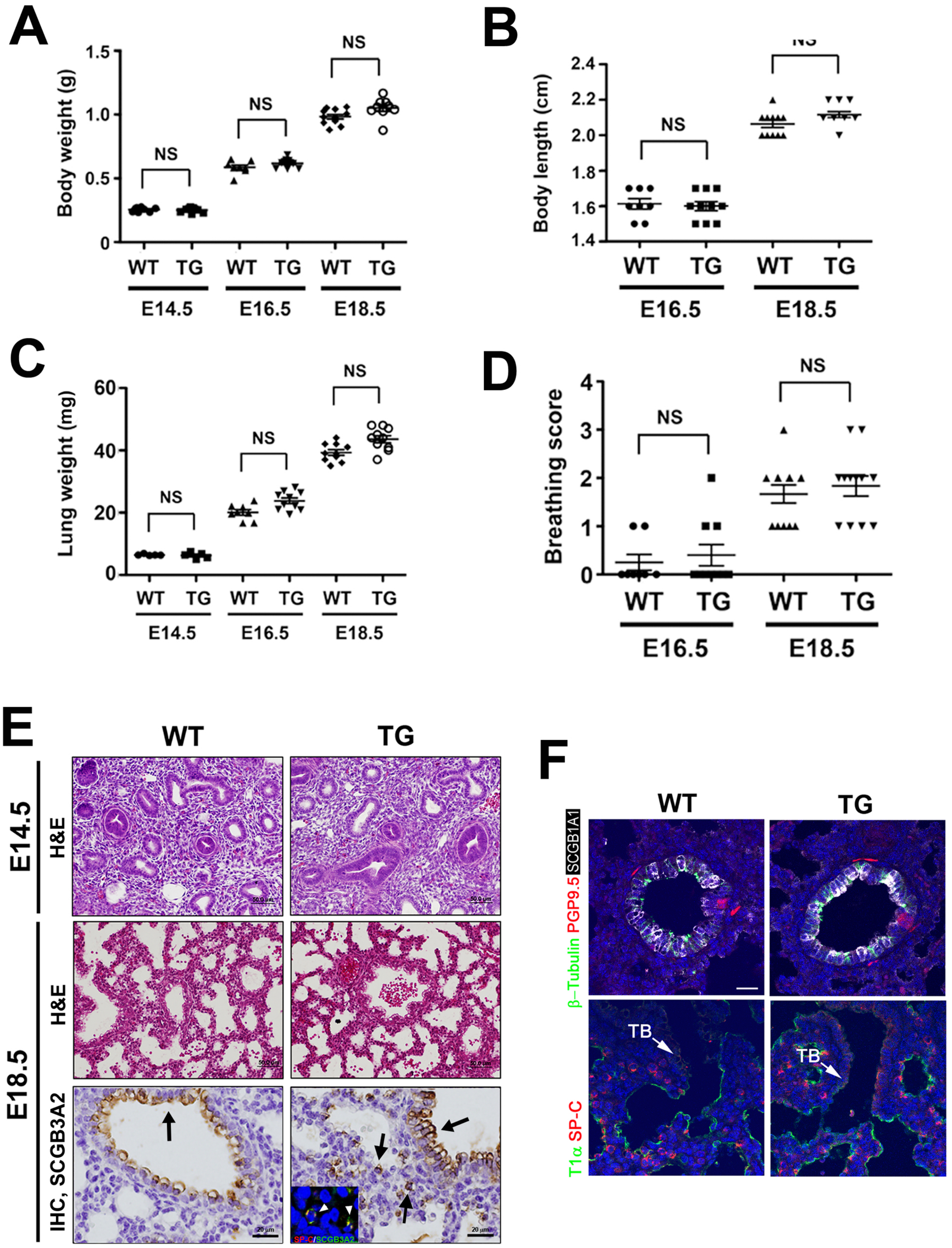

A
NS
NS
NS
Body weight (g)
1.5
1.0
0.5
0
WT TG WT TG WT TG
E14.5 E16.5 E18.5
B
NS
NS
Body length (cm)
2.4
2.2
2.0
1.8
1.6
1.4
WT TG WT TG
E16.5 E18.5
C
NS
NS
NS
Lung weight (mg)
60
40
20
0
WT TG WT TG WT TG
E14.5 E16.5 E18.5
D
NS
NS
Breathing score
4
3
2
1
0
WT TG WT TG
E16.5 E18.5
E
WT
TG
E14.5
H&E
E18.5
H&E
IHC, SCGB3A2
SP-C/SCGB3A2
F
WT
TG
β-Tubulin PGP9.5 SCGB1A1
T1α SP-C
TB
TB

Figure S3

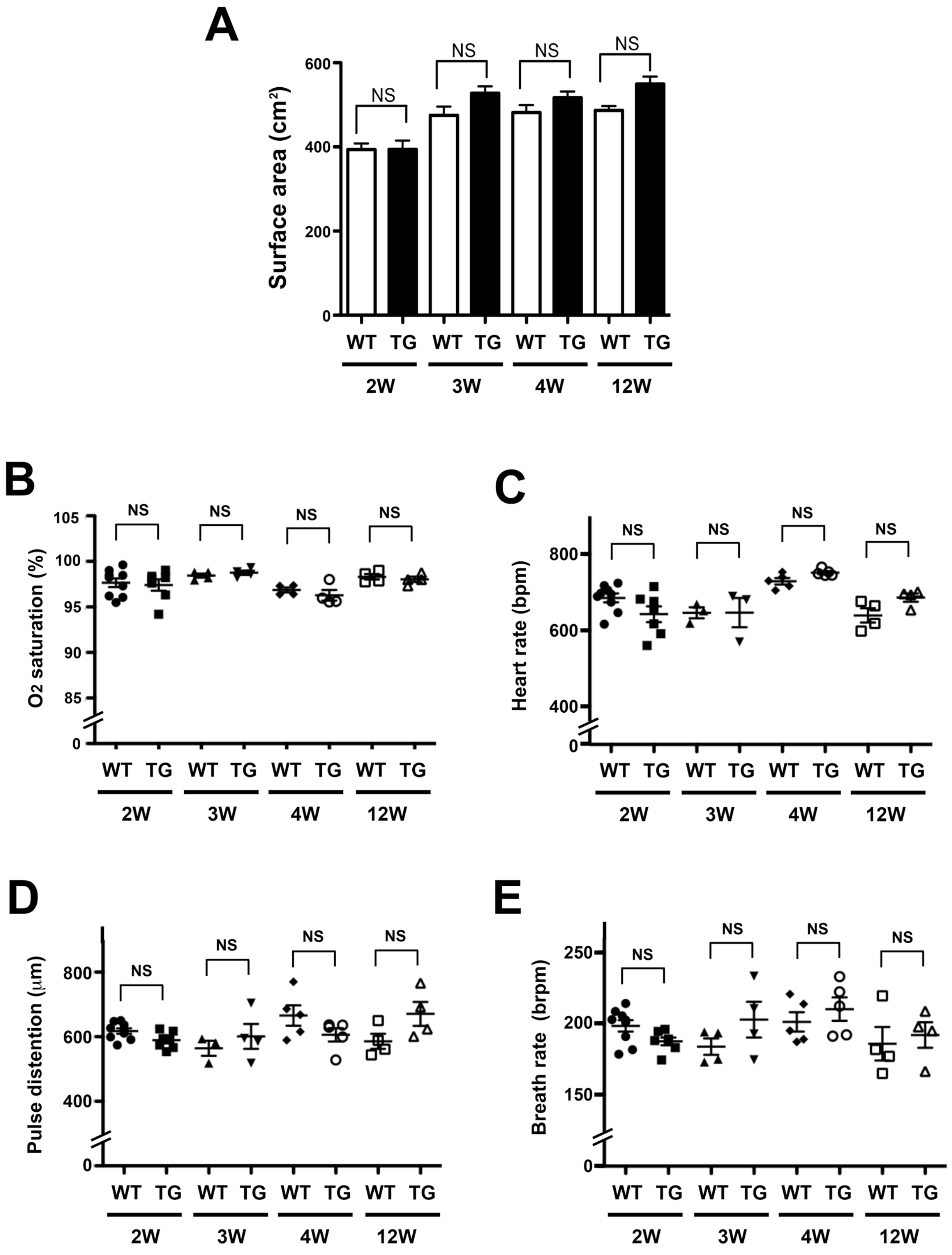

A
Surface area (cm²)
600
400
200
0
NS
NS
NS
NS
WT TG WT TG WT TG WT TG
2W 3W 4W 12W
B
O2 saturation (%)
105
100
95
90
85
0
NS
NS
NS
NS
WT TG WT TG WT TG WT TG
2W 3W 4W 12W
C
Heart rate (bpm)
800
600
400
0
NS
NS
NS
NS
WT TG WT TG WT TG WT TG
2W 3W 4W 12W
D
Pulse distention (µm)
800
600
400
0
NS
NS
NS
NS
WT TG WT TG WT TG WT TG
2W 3W 4W 12W
E
Breath rate (brpm)
250
200
150
0
NS
NS
NS
NS
WT TG WT TG WT TG WT TG
2W 3W 4W 12W

Figure S4

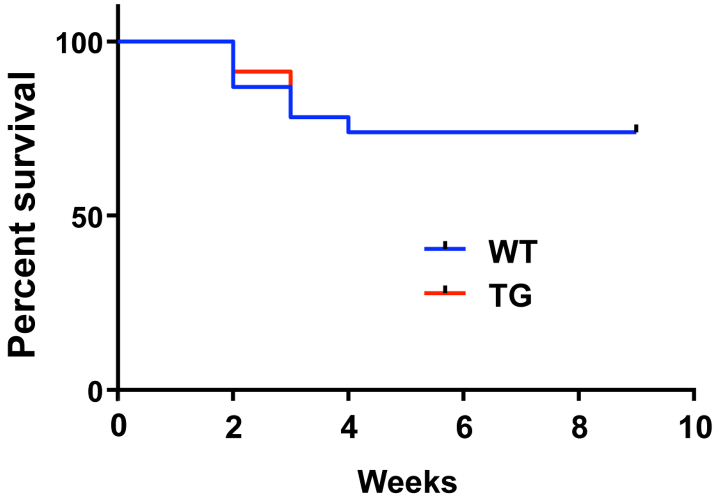

Figure S5

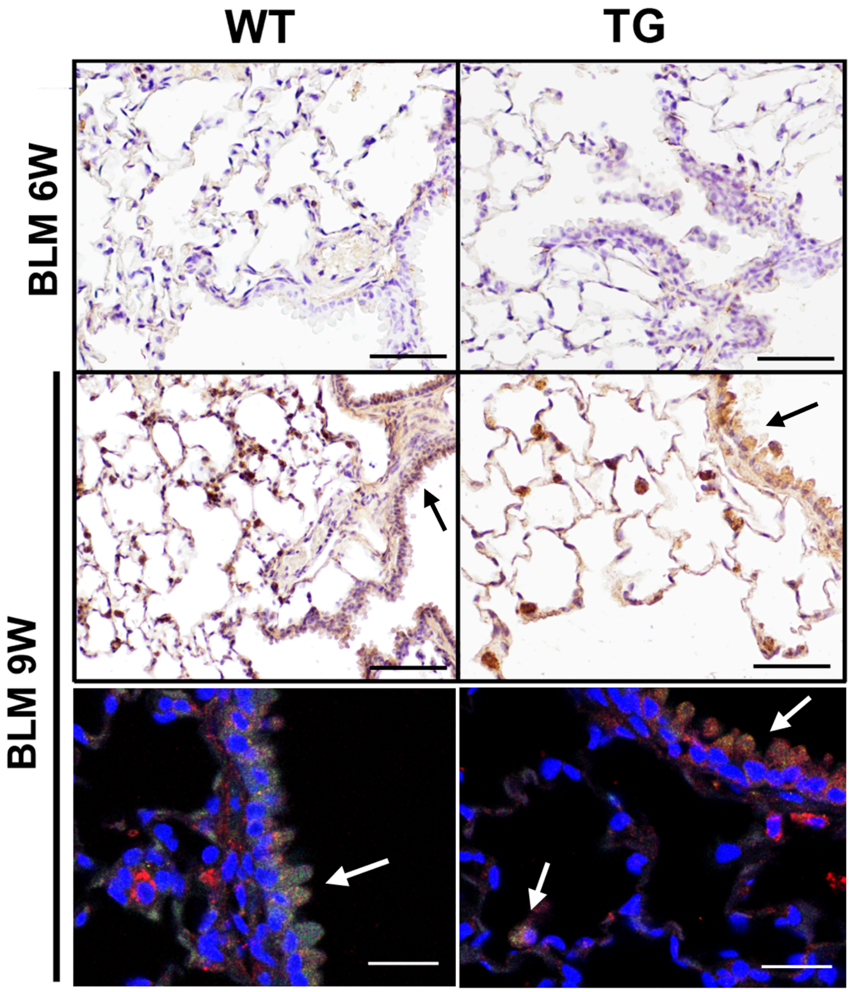

Supplement: Additional file 1: Figure S1. — Scanning electron microscopy of wild-type (WT) and Scgb3a2-transgenic mouse (TG) airways. Figure S2. Characterization of Scgb3a2-transgenic embryo lungs. Figure S3. Lung morphometric analysis. Figure S4. Survival curve of wild-type (WT) and Scgb3a2-transgenic mice (TG) after BLM treatment. Figure S5. SP-C expression in wild-type (WT) and Scgb3a2-transgenic mouse (TG) lungs at 6 and 9 weeks post-BLM administration. [file 12890_2015_65_MOESM1_ESM.pdf]
